# The Cure of Consumption

**Published:** 1890-06

**Authors:** 


### THE CURE OF CONSUMPTION.

The Boston *Herald* recently mentioned the case of the father of a highly respected Boston physician who, at a somewhat late stage of consumption, when so weak as to be hardly able to walk, took his horse and chaise, and with a friend as a companion, journeyed from place to place for several weeks, and returned practically cured.

What was the secret of that cure? Clearly not medical drugs. Yet it is just these that the consumptive generally relies on almost exclusively, taking the prescription daily in his cushioned chair, until he is lifted to his bed, to wear out the little remnant of his life.

From first to last his medicines have merely, or mainly, made him feel more comfortable, while the disease has steadily progressed to the fatal end.

In the case of the wiser man mentioned above, pure air was the chief element of cure; and the pure air was kept pure, for, while his own breathing tended to infect it, he was constantly leaving the infection behind him. To the consumptive, pure air is always the first requisite, and the main value of winter resorts is in their allowing the patient to spend so large a part of his time out of doors.

A distinguished physician once said that if he were attacked with consumption, he would build a shed to his house and sleep in it. Thus, through the free circulation, he would avoid the constant re-breathing of his own infected breath.

But in the case under consideration, the breathing of pure air was not the only advantage of the course pursued. The man's daily travel gave him a gentle exercise suited to his condition. Disease can be thrown off only by the activity of the various life processes—digestion, assimilation, secretion, excretion, and the many chemical and vital changes.

Now, it is an established fact that a certain amount of physical exercise is essential to such activity of the vital processes. In the case of the sick, it is especially essential, since the system must be daily ridding itself of morbid matter, and be making unwonted draughts on its recuperative power.

In some cases, a horse's back would be better than the chaise ; but the exercise must be suited to the particular case. It must never be fatiguing.

Another element of the cure was the change of scene. It is a great help to have the mind diverted from one's symptoms, and pleasantly taken up with new surroundings, as far as possible removed from ordinary business cares and the accustomed routine of thought.

But consumption is a disease which specially demands aid in keeping up a feeble appetite. The invalid must be enabled to eat a liberal supply of easily digested food, and the above conditions all tend to serve him in this direction.

